# Can adalimumab prevent from acute effects of lipopolysaccharide induced renal injury in rats?

**DOI:** 10.1590/acb394624

**Published:** 2024-09-02

**Authors:** Nuket Özkavruk Eliyatkın, Akif İşlek, Selim Durmaz, Fevzi Ayyıldız, Ömer Rahman

**Affiliations:** 1 İzmir Katip Çelebi University – Faculty of Medicine – Department of Pathology – İzmir – Turkey.; 2 Acıbadem Eskişehir Hospital – Department of Otorhinolaryngology – Eskisehir – Turkey.; 3 Aydın Adnan Menderes University – Faculty of Medicine – Department of Cardiovascular Surgery – Aydın – Turkey.; 4 Burdur State Hospital – Department of Cardiovascular Surgery – Burdur – Turkey.

**Keywords:** Adalimumab, Lipopolysaccharides, Acute Kidney Injury

## Abstract

**Purpose:**

Lipopolysaccharides is well-known in the acute renal injury process. It causes widespread activation of inflammatory cascades. Tumor necrosis factor (TNF)-α and interleukin (Il)-6 are essential proinflammatory cytokines that can induce the production of other cytokines in host response. Adalimumab suppresses TNF-α, IL-1β, and IL-6. We aimed to evaluate whether adalimumab would prevent the toxicity of lipopolysaccharide on the rat renal tissue.

**Methods:**

Adult female Wistar rats were divided into four groups. To the control group, only intraperitoneal saline injection procedure was carried out. For adalimumab group, adalimumab was injected at a dose for two days. For lipopolysaccharide group, animals were injected with lipopolysaccharide (a dose). For lipopolysaccharide-adalimumab group, animals were given adalimumab treatment before the injection of lipopolysaccharide. Histopathological changes and immunohistochemical analysis for TNF-α and IL-6 were determined.

**Results:**

The pathological changes and immunohistochemical staining for TNF-α or IL-6 were similar for control and adalimumab groups (*p* > 0.05). The lipopolysaccharide group had significantly higher distorted features in the renal tissues (*p* < 0.001), and also significantly prominent immunohistochemical staining for TNF-α or IL-6 (0.003), compared to the control group. No severe pathological feature was detected in the lipopolysaccharide-adalimumab group, but moderate necrosis was found in all cases (*p* = 0.003). TNF-α staining and IL-6 staining in the lipopolysaccharide group was found to significantly prominent compared to lipopolysaccharide-adalimumab group (*p* = 0.013).

**Conclusions:**

Because of its anti-inflammatory property, adalimumab pretreatment may have protective effects on experimental kidney injury. Adalimumab could be considered as a protective agent to acute effects of lipopolysaccharide induced renal injury.

## Introduction

Acute renal injury (ARI) is one of the most frequent diseases in the intensive care unit, with a rate of approximately 10–15%. Clinically, ARI occurs with an increase in serum creatinine and/or decrease in urine output, also pathological morphology of disease characterized by injury of renal tubular vacuolar changes, including degeneration and necrosis of tubular epithelial cells and diaphanous tubular cast, hemorrhage/congestion, necrosis, inflammatory cell infiltration, glomerular changes^1^^,^ 2. Systemic inflammation or sepsis has been known to have a major role in the development of ARI, or ARI frequently facilitates sepsis^2^^,^ 3.

Gram-negative bacteria and their cell wall component lipopolysaccharides (LPS) have been well-known and studied pathophysiology in the ARI process. The recognition of LPS by tubular epithelial cells and endothelial cells in the kidney requires a specific receptor, which belongs to the Toll-like receptor (TLR) family of proteins, called TLR4, and two carrier proteins, namely the LPS-binding protein (LBP) and the cluster of differentiation 14 (CD14)^4^^,^ 5.

The endotoxin in the LPS component is inactive as long as it remains in the cell membrane, but various stimuli including sepsis, severe trauma, and major surgery procedure cause the endotoxin to be released by separating it from the cell membrane and initiating the sepsis/endotoxemia sequence of events. Thus, it causes widespread activation of inflammatory cascades. Previous studies have reported that gram-negative bacterial infection is the main cause of systemic inflammation/systemic inflammatory response syndrome^2^^,^ 4 ^,^ 6 ^–^ 8. The increased level of circulating proinflammatory cytokines due to the endotoxin load also contributes to ARI^1^^,^ 4.

Tumor necrosis factor alpha (TNF-α) and interleukin-6 (IL-6) are essential proinflammatory cytokines that can induce the production of other cytokines in host response during ARI^4^^–^ 6 ^,^ 9 ^–^ 13. Adalimumab (Ada) is a potent inhibitor of TNF-α. It suppresses TNF-α, IL-1β, and IL-6, leading to reduction or inhibition of the inflammatory process^14^^,^ 15. Ada is commonly used in various inflammatory/immune-mediated diseases / rheumatological diseases such as rheumatoid arthritis and ankylosing spondylitis, inflammatory bowel disease, psoriatic arthritis^16^. It blocks the interaction of TNF-α with the cell surface receptors, to modify the inflammatory responses^16^^,^ 17.

In our previous rat study, we demonstrated the protective effect of Ada on endotoxin-induced cardiac damage^6^. It was thought that this effect might be related to Ada’s reduction of cytokine release. Similarly, we thought that we could evaluate the results of Ada application in rats with endotoxin-induced renal damage. In literature, Ada has been demonstrated to blockage the release of pro‐inflammatory cytokines like TNF‐α and to be protective against renal ischemia/reperfusion in a rat model of abdominal aorta cross-clamping^8^. However, according to our knowledge, Ada has been not investigated in endotoxin induced renal damage in rats.

The aim of our experimental study was to investigate the toxicity of LPS on rat renal tissues and the protective effect of Ada in kidney damage caused by LPS.

## Methods

### Chemicals

LPS (*Escherichia coli* 0111:B4) were obtained from Sigma Aldrich, Deisenhofen, Germany. Ada (Humira) was supplied by Vetter Pharma-Fertigung GmBH&Co.KG, Ravensburg, Germany. The doses used for this study were 5 mg/kg (i.p) for LPS and 10 mg/kg/day (i.p.) for Ada.

### Animal grouping and treatment, study protocol

The experimental design and protocol were approved by the Animal Care Committee of Adnan Menderes University (approval number: 64583101/2023/17). The animal care was conducted in accordance with the National Institute of Health’s Guide for the Care and Use of Laboratory Animals. The animals used in the experiment were obtained from the Aydın Adnan Menderes University Experimental Animals Research and Production Laboratory.

Adult female Wistar rats (approximately weighing 170–270 g) were used in this study. Rats fed a standard rat chow diet and water ad libitum and were kept in cages in a controlled temperature (22°C ± 2°C) and humidity (45–50%) with a 12-h dark-light cycle and acclimatized for a week before the study.

Rats were divided into four following groups, each consisting of seven rats:

- Control (C): animals were injected once a day with intraperitoneal (i.p.) 0.9% saline for two days. After the injection, they were returned to cages and allowed standard rat chow and water ad libitum for 24 h;
- Adalimumab (Ada): adalimumab (Humira, Vetter Pharma-Fertigung GmBH&Co.KG, Ravensburg, Germany) was injected at the dose of 10 mg/kg/day (i.p.) for two days. The animals were returned to cages and allowed standard rat chow and water ad libitum for 24 h after last injection;
- Lipopolysaccharide (LPS): animals were injected with lipopolysaccharide (*Escherichia coli* 0111:B4 Sigma, Deisenhofen, Germany) with the dose of 5 mg/kg (i.p.), then returned to their cages and were allowed standard rat chow and water ad libitum for 24 h;
- Lipopolysaccharide + adalimumab (LPS+Ada): animals were given ada treatment as already described before the injection of lipopolysaccharide. Then, they were returned to cages and allowed standard rat chow and water ad libitum for 24 h after the last injection.

Our study was conducted taking into account the protocol we applied in our previous study protocol in terms of drug administration doses^6^.

### Renal tissue sampling

In each group, 24 hours later the last injection, animals were anesthetized with a combination of ketamine hydrochloride and xylazine injection (i.p.). Then, the rats were sacrificed by cervical dislocation, and, immediately after that, the kidney was dissected free from the surrounding tissues, harvested for histopathological examination and fixed in 10% phosphate-buffered formalin.

### Histological study

#### Histopathological evaluation

**Figure 1:**
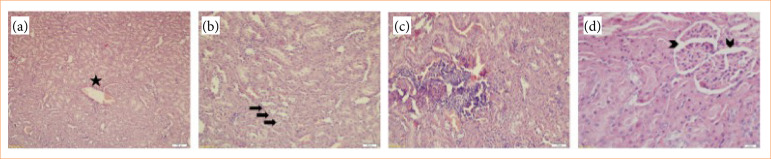
The pathological changes in lipopolysaccharides group. **(a)** Congestion (star); **(b)** tubular vacuolar degeneration (arrows); **(c)** inflammatory cell infiltration; **(d)** glomerular changes (arrowheads).

Source: Elaborated by the authors.

Harvested renal tissues were fixed in 10% formalin for 24 h, then dehydrated in a graded ethanol series, cleared in xylene, and embedded in paraffin. In addition, 4-μm thick serial sections were obtained and put on poly-L-lysine slides. All groups were stained with hematoxylin-eosin (H&E) staining to gain a morphological overview of the tissue and its structure. The pathological changes including hemorrhage/congestion, necrosis, tubular vacuolar degeneration, inflammatory cell infiltration, glomerular changes in the renal tissues were evaluated under an Olympus BX-51 light microscope (Olympus BX-51, Tokyo, Japan) by a blinded pathologist. The preparates in all groups were plotted as none (-), mild (+), moderate (++), and severe (+++) damage (Fig. 1).

#### Immunohistochemical analysis

Sections of 3–4-μm thickness were cut for the immunohistochemical staining. The sections were rinsed in deionized water, and antigen retrieval was performed by incubation in a 10% citrate buffer (pH = 6.0) at 95°C for 5 min, and then cooled to room temperature for 20 min. The sections were incubated in 3% H_2_O_2_ for 10 min and rinsed in a phosphate-buffered saline (PBS). An antipolyvalent HRP antibody labeling kit (Thermo Scientific, United States of America) was used for the following steps.

**Figure 2:**
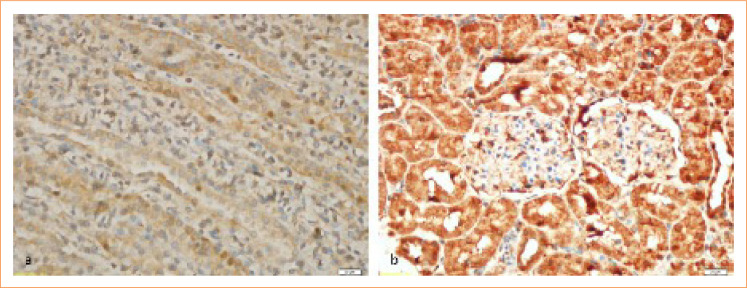
Immunohistochemical staining for tumor necrosis factor α. **(a)** Control group, **(b)** lipopolysaccharides group.

Source: Elaborated by the authors.

**Figure 3:**
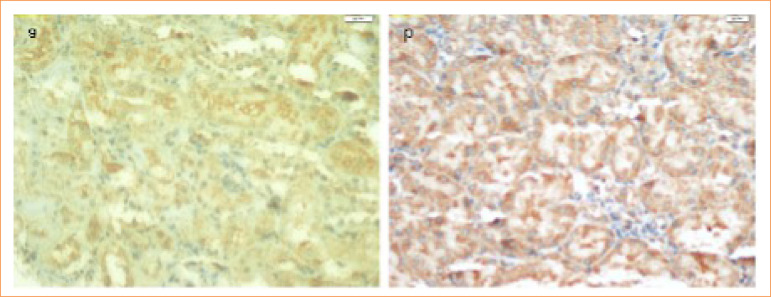
Immunohistochemical staining for interleukin-6. **(a)** Control group, **(b)** lipopolysaccharides group.

Source: Elaborated by the authors.

To reduce nonspecific staining, the sections were pretreated with normal block serum for 10 min. Each slide was put in certain dilution containing primary antibody (TNF-α 1 μg/mL) for 75 min before staining them by anti-TNF-α antibody (sc: 52746, Santa Cruz Biotechnology, United States of America) and anti IL-6 antibody (sc: 130326, Santa Cruz Biotechnology, United States of America). While diaminobenzidine solution was used as a chromogen, a counterstain Mayer’s hematoxylin was used for 3–5 min. PBS was used as a negative was photographed. After being covered with appropriate covering materials, all the preparations were evaluated. They were subdivided into four categories depending on the percentage of immunopositivity of the tissue as (+) for mild, (++) for moderate, (+++) for severe, and (++++) for very severe. A thorough evaluation was made to the results of the statistical comparisons that were obtained during, between and within group evaluations (Figs. 2 and 3).

### Statistical analysis

Statistical analysis was performed using the IBM Statistical Package for the Social Sciences version 22.0 program (IBM Corp., Armonk, NY, United States of America). The normal distribution analysis of the data was evaluated with the Shapiro-Wilk’s test. The χ^2^ test was used to compare the variables between the groups. In statistical analysis, the significance limit was accepted as *p* < 0.05.

## Results

### General results of histopathological and immunohistochemical staining

No pathological findings were found in control and Ada groups. Glomerulus and Bowman’s capsul were seen in the normal histologic structure. Also, no degeneration was found in kidney tubules of rats. After LPS treatment, hemorrhage/congestion, necrosis, tubular degeneration, inflammatory cell infiltration, glomerular changes (glomerular lobulation and dilatation of Bowman’s space) were detected in the kidneys of the rats according to the light microscopic examination. LPS and LPS+Ada groups displayed pathological deformations of the epithelial structure of tubules, glomerular changes, hemorrhage/congestion, necrosis, inflammatory cell infiltration, but the histopathologic changes observed in LPS group were more pronounced than those seen in the LPS group.

In our study, immunohistochemical TNF-α and IL-6 method was used to evaluate proinflammatory cytokines in kidney tissues of C, Ada, LPS, LPS+Ada groups. The results of immunostaining with TNF-α and IL-6 were similar in the kidney of the control group and Ada group. Both TNF-α and IL-6 immunohistochemical staining results in all rats kidney tissues showed very intense staining for the LPS group. Although TNF-α and IL-6 immunostaining were observed in the LPS+Ada treatment group, the intensity of this staining was not strong in any kidney tissue.

### Statistical results of histopathological and immunohistochemical staining

**Figure 4:**
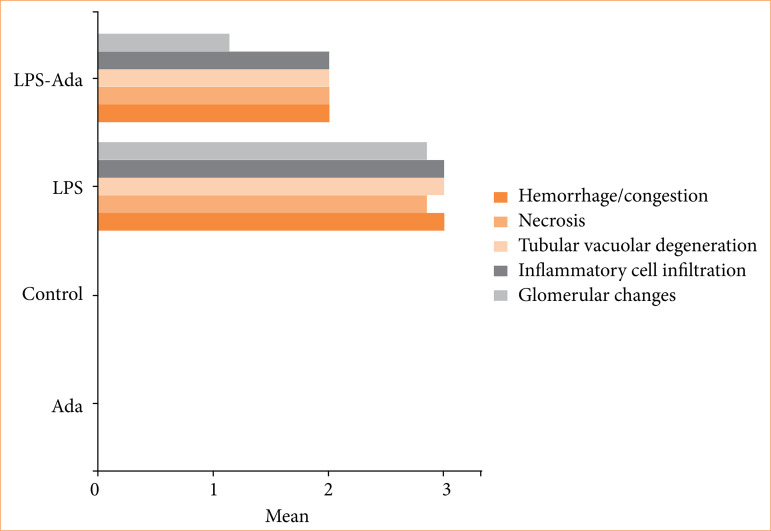
The results of histopathological injury scores.

LPS: lipopolysaccharides; Ada: adalimumab. Source: Elaborated by the authors.

**Figure 5:**
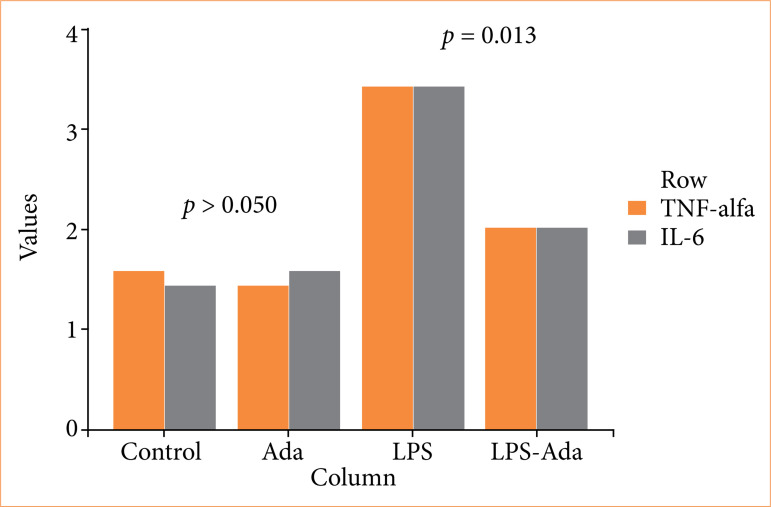
The results of immunohistochemical staining for TNF-α and IL-6 scores.

LPS: lipopolysaccharides; Ada: adalimumab; TNF-α: tumor necrosis factor-α; IL-6: interleukin-6. Source: Elaborated by the authors.

The pathological changes in the renal tissues including hemorrhage/congestion, necrosis, tubular vacuolar degeneration, inflammatory cell infiltration, glomerular changes, and immunohistochemical staining for TNF-α or IL-6 were similar for control and Ada groups (*p* > 0.05, χ^2^ test). The LPS group had significantly higher distorted features in the renal tissues for hemorrhage/congestion (*p* < 0.001), necrosis (*p* < 0.001), tubular vacuolar degeneration (*p* < 0.001), inflammatory cell infiltration (*p* < 0.001), glomerular changes (*p* < 0.001), or also significantly prominent immunohistochemical staining for TNF-α (0.003) or IL-6 (0.003), compared to the control group (χ^2^ test). Severe hemorrhage/congestion, tubular vacuolar degeneration, and inflammatory cell infiltration were detected in all cases in the LPS group, while it was found to be moderate (*p* < 0.001, χ^2^ test) in all cases in the LPS+Ada group. Similarly, severe necrosis was detected in the LPS group with a rate of 85.7%, while no severe necrosis was detected in the LPS+Ada group. Also, no severe pathological feature was detected in the LPS+Ada group, but moderate necrosis was found in all cases (*p* = 0.003). While severe glomerular distortion was detected in the LPS group with a rate of 85.7%, it occurred as mild with a rate of 85.7% in the LPS+Ada group (*p* = 0.002, χ^2^ test). TNF-α staining in the LPS group was found to be very severe in 42.9% of the cases and severe in 57.1% of the cases, but it was found moderate staining in 71.4% of the cases, mild in 14.3%, and severe in only 14.3% of the cases in the LPS-Ada group (*p* = 0.013). Parallelly, IL-6 staining was classified as very severe or severe in all cases in the LPS group, but in the LPS-Ada group it was found that mild or moderate staining was detected in 85.7% of cases (*p* = 0.013, χ^2^ test). The results of histopathological injury scores are shown in Fig. 4, and the results of immunohistochemical staining for TNF-α and IL-6 scores are shown in Fig. 5.

## Discussion

In our study, the pathological changes in the renal tissues including hemorrhage/congestion, necrosis, tubular vacuolar degeneration, inflammatory cell infiltration, glomerular changes, and immunohistochemical staining for TNF-α or IL-6 were similar for control and Ada groups. The LPS group had significantly higher distorted features in the renal tissues, or also significantly prominent immunohistochemical staining for TNF-α or IL-6, compared to the C group. In the LPS-Ada group, renal pathological features and TNF-a / IL-6 staining were scored significantly lower than in the LPS group. These findings indicated that the properties of LPS endotoxin on renal structures were significantly inhibited by Ada, even if not completely.

Major causes of ARI are mainly classified under the headings of sepsis, renal hypoperfusion and toxicity. Therefore, prevention of ARI or management of the disease varies according to these etiological causes^1^^,^ 4 ^,^ 18. Cure et al.^19^ demonstrated the preventive effect of Ada in the ischemia-reperfusion (I/R) animal model. They showed that the trigger of renal I/R injury is the production of proinflammatory cytokines, such as TNF-α, IL-1β, and IL-6, also similar to LPS-induced renal injury. Also, they detected that proinflammatory cytokines were suppressed significantly in the Ada treatment group. Additionally, they found that the histopathological features of the Ada treatment group revealed less damage and lower TNF-α activity. So, they supported that Ada may prevent reperfusion injury by suppressing the release of TNF-α, IL-1β, and IL-6^19^.

Similar to the presented study, Durmaz et al.6 showed the preventive properties of Ada in an experimental study including LPS-induced myocardial injury. They indicated that Ada treatment significantly reduces circulating proinflammatory and also a significant recuperation in histopathologic signs of myocardial injury. Furthermore, they observed that the Ada treatment at the selected dose in rats did not cause any cardiac injury.

There is no study in the literature examining the pharmacological effects of Ada in any LPS-associated ARI model. Recently, a case report discussed Ada immunotherapy for a patient diagnosed with Crohn’s disease who had acute kidney injury caused by COVID-19 sepsis. Although the positive reports for the beneficial effect of Ada in LPS-related cardiotoxicity, or I/R-associated nephropathy, Ada had a risk to induce interstitial nephritis in a mice model, also in a few case reports^20^^,^ 21. On the other hand, no pathology was detected histopathologically or immunohistochemically in animals with Ada administered in this study, and the findings were the same as in the C group. LPS-related injury is a model that can damage to many different tissues and is widely used in the experimental studies. Various agents that may protect against tissue damage caused by LPS are being investigated.

These also include various minerals and vitamins. Ilçe et al.^22^ evaluated the acute toxicity of LPS on rat renal cells as histology, formation of apoptosis, changes in antioxidant enzyme acivities, malondialdehyde levels, and DNA structure and investigated the protective effect either alone or as a combination of selenium and vitamin E on kidney damage caused by LPS^22^. They showed that the selenium and vitamin E were reduced significantly with LPS induced nephrotoxicity in rats, but not completely protected.

Since LPS causes damage in different tissues, the protective effect of Ada on different organs has been investigated through experimental studies. Sarıoğlu et al.^23^ showed Ada and/or tocilizumab have a more potent anti-inflammatory effect on lung injury than the steroid. The protective effects of Ada have been investigated over the years in a variety of disease or injury conditions (including experimental models)^24^^–^ 27.

According to our knowledge, the protective effect of Ada has been not investigated in endotoxin induced renal damage in rats. As a result of this literature research, we evaluated histopathologically the effects of endotoxin induced-renal damage in our experimentel study. In addition, immunohistochemical staining was performed to determine tissue levels of TNF and IL, which are basic cytokines, and we discussed the possible prophylactic potential and/or the protective effects.

## Conclusion

Even in nowaday’s conditions, sepsis is among the areas of interest of modern medicine. Because sepsis mortality is high and an increase in the number of cases is reported every year, the results of relevant studies on sepsis (both preclinical and clinical type) have not yet achieved the desired success. The main treatment for sepsis is antimicrobial agents. Supportive treatments also contribute. However, there are few agents with proven effectiveness. Sepsis has a very complicated physiopathological process. Nowaday’s progress in molecular biology has helped the field of sepsis become more understandable. Despite this, there is a need for agents that can be effective and protective in the pathophysiological processes in the development of sepsis. More studies should be done on this subject.

In our study, the positive effects of Ada biological agents on LPS-associated ARI were shown, albeit partially, histologically and immunohistochemically. To our knowledge, this study is the first experimental controlled study demonstrating the effect of Ada in LPS-induced ARI. In future studies, studies that include the nephrotoxic effects of Ada molecule on the kidney, as well as its anti-inflammatory effects, will yield more valid results.

### Limitations of the study

The limitations of our study are that the results obtained from animal studies cannot be generalized directly to humans. We may have obtained positive results for these drugs in our animal study, but randomized controlled studies are needed to see their effects in humans. Ada is the first anti-TNF-α monoclonal antibody obtained from humans. It is approved by the Food and Drug Administration for clinical use only in treatment of some autuimmune diseases such as Crohn’s disease, rheumatoid arthritis, ankylosing spondylitis. More studies should be done on this subject.

We could not evaluate levels of urine and circulating levels of TNF-α and IL-6. Instead of these evaluations, we performed immunohistochemical analysis.

## Data Availability

All datasets were generated or analyzed during the current study.

## References

[1] Ronco C, Bellomo R, Kellum JA (2019). Acute kidney injury. Lancet.

[2] Peerapornratana S, Manrique-Caballero CL, Gómez H, Kellum JA (2019). Acute kidney injury from sepsis: current concepts, epidemiology, pathophysiology, prevention and treatment. Kidney Int.

[3] Yoon SY, Kim JS, Jeong KH, Kim SK (2022). Acute Kidney Injury: Biomarker-Guided Diagnosis and Management. Med. 2022.

[4] Stasi A, Intini A, Divella C, Franzin R, Montemurno E, Grandaliano G, Ronco C, Fiaccadori E, Pertosa GB, Gesualdo L, Castellano G (2017). Emerging role of Lipopolysaccharide binding protein in sepsis-induced acute kidney injury. Nephrol Dial Transplant.

[5] El-Achkar TM, Hosein M, Dagher PC (2008). Pathways of renal injury in systemic gram-negative sepsis. Eur J Clin Invest.

[6] Durmaz S, Kurtoğlu T, Barbarus E, Eliyatkın N, Yılmaz M (2020). TNF-alpha inhibitor adalimumab attenuates endotoxin induced cardiac damage in rats. Acta Cir Bras.

[7] Castellheim A, Brekke O-L, Espevik T, Harboe M, Mollnes TE. (2009). Innate immune responses to danger signals in systemic inflammatory response syndrome and sepsis. Scand J Immunol.

[8] Cavaillon J-M, Annane D (2006). Compartmentalization of the inflammatory response in sepsis and SIRS. J Endotoxin Res..

[9] Zhang Y, Liang D, Dong L, Ge X, Xu F, Chen W, Dai Y, Li H, Zou P, Yang S, Liang G (2015). Anti-inflammatory effects of novel curcumin analogs in experimental acute lung injury. Respir Res..

[10] Du ZA, Sun MN, Hu ZS (2018). Saikosaponin an Ameliorates LPS-Induced Acute Lung Injury in Mice. Inflammation.

[11] Bhatia M, Moochhala S (2004). Role of inflammatory mediators in the pathophysiology of acute respiratory distress syndrome. J Pathology.

[12] Lei J, Wei Y, Song P, Li Y, Zhang T, Feng Q, Xu G (2018). Cordycepin inhibits LPS-induced acute lung injury by inhibiting inflammation and oxidative stress. Eur J Pharmacol.

[13] Akdis M, Burgler S, Crameri R, Eiwegger T, Fujita H, Gomez E, Klunker S, Meyer N, O’Mahony L, Palomares O, Rhyner C, Ouaked N, Schaffartzik A, Van De, Zeller S, Zimmermann M, Akdis CA. (2011). Interleukins, from 1 to 37, and interferon-γ: receptors, functions, and roles in diseases. J Allergy Clin Immunol.

[14] McGovern JL, Nguyen DX, Notley CA, Mauri C, Isenberg DA, Ehrenstein MR (2012). Th17 cells are restrained by Treg cells via the inhibition of interleukin-6 in patients with rheumatoid arthritis responding to anti-tumor necrosis factor antibody therapy. Arthritis Rheum.

[15] Kayakabe K, Kuroiwa T, Sakurai N, Ikeuchi H, Sakairi T, Kaneko Y, Maeshima A, Hiromura K, Nojima Y (2012). Interleukin-1β measurement in stimulated whole blood cultures is useful to predict response to anti-TNF therapies in rheumatoid arthritis. Rheumatology (Oxford).

[16] Burmester GR, Panaccione R, Gordon KB, McIlraith MJ, Lacerda AP (2013). Adalimumab: long-term safety in 23,458 patients from global clinical trials in rheumatoid arthritis, juvenile idiopathic arthritis, ankylosing spondylitis, psoriatic arthritis, psoriasis and Crohn’s disease. Ann Rheum Dis.

[17] Javed Q, Murtaza I (2013). Therapeutic potential of tumour necrosis factor-alpha antagonists in patients with chronic heart failure. Hear Lung Circ.

[18] Virzì GM, Clementi A, Brocca A, Ronco C (2017). Endotoxin Effects on Cardiac and Renal Functions and Cardiorenal Syndromes. Blood Purif.

[19] Cure MC, Cure E, Kalkan Y, Tumkaya L, Aydin I, Kirbas A, Efe H, Kurt A, Yuce S (2016). The protective effect of Adalimumab on renal injury in a model of abdominal aorta cross-clamping. Adv Clin Exp Med.

[20] Mogairen SA (2017). Adalimumab induced chronic interstitial nephritis: a controlled blinded trial in mice. Int J Clin Rheumtol.

[21] Sandys V, Moloney B, Lane L, Qazi J, Doyle B, Barry M, Leavey S, Conlon P (2018). Granulomatous interstitial nephritis secondary to adalimumab therapy. Clin Kidney J.

[22] Ilçe F, Gök G, Pandir D (2019). Acute effects of lipopolysaccharide (LPS) in kidney of rats and preventive role of vitamin E and sodium selenite. Human and Experimental Toxicology.

[23] Sarıoğlu N, Sunay FB, Yay A, Korkut O, Erel F, Hişmioğulları AA, Köse M, Yalçın B (2021). Antiinflammatory effects of adalimumab, tocilizumab, and steroid on lipopolysaccharide-induced lung injury. Turk J Med Sci.

[24] Pergel A, Tümkaya L, Demiral G, Çolakoğlu MK, Kalcan S, Özdemir A, Mercantepe T, Erdivanlı B, Yılmaz A (2020). The protective effects of adalimumab on intestinal injury induced with infrarenal aortic occlusion. Ulus Travma Acil Cerrahi Derg.

[25] Kocamaz H, Özdemir ÖM, Türk NŞ, Enli Y, Şahin B, Ergin H (2021). Dose-dependent effects of adalimumab in neonatal rats with hypoxia/reoxygenation-induced intestinal damage. Bosn J Basic Med Sci.

[26] Cure E, Cumhur M, Tumkaya L, Kalkan Y, Aydin I, Kirbas A, Yilmaz I, Kirbas A, Yücel A (2014). Adalimumab ameliorates abdominal aorta cross clamping which induced liver injury in rats. Biomed Res Int.

[27] Kaplan S, Kırıcı P, Türk A (2022). The effects of adalimumab on the rat autotransplantation endometriosis model: A placebo-controlled randomized study. Adv Clin Exp Med.

